# Bovine Milk-Derived Extracellular Vesicles Ameliorate Steatohepatitis by Restoring Gut Barrier in CDA-HFD-Fed Mice

**DOI:** 10.3390/ijms27146485

**Published:** 2026-07-21

**Authors:** Tatsuya Nakatani, Shinya Sato, Kosuke Kaji, Hiroki Kachi, Naoki Nishimura, Masafumi Oyama, Jun-ichi Hanatani, Satoshi Iwai, Soichi Takeda, Norihisa Nishimura, Koh Kitagawa, Tadashi Namisaki, Hitoshi Yoshiji

**Affiliations:** Department of Gastroenterology, Nara Medical University, 840 Shijo-cho, Kashihara 634-8522, Nara, Japan; k182324@naramed-u.ac.jp (T.N.); kajik@naramed-u.ac.jp (K.K.); kachi@naramed-u.ac.jp (H.K.); k147051@naramed-u.ac.jp (N.N.); k184066@naramed-u.ac.jp (M.O.); k136081@naramed-u.ac.jp (J.-i.H.); satoshi181@naramed-u.ac.jp (S.I.); souitit@naramed-u.ac.jp (S.T.); nishimuran@naramed-u.ac.jp (N.N.); kitagawa@naramed-u.ac.jp (K.K.); tadashin@naramed-u.ac.jp (T.N.); yoshijih@naramed-u.ac.jp (H.Y.)

**Keywords:** extracellular vesicles, gut–liver axis, intestinal barrier, metabolic dysfunction-associated steatohepatitis, microbiota

## Abstract

Gut barrier dysfunction and portal endotoxemia contribute to metabolic dysfunction-associated steatohepatitis (MASH) progression through activation of hepatic inflammatory signaling. Milk-derived extracellular vesicles (EVs) contain bioactive microRNAs and have recently attracted attention as modulators of intestinal homeostasis. This study investigated the effect of bovine milk-derived extracellular vesicles (B-mEVs) in ameliorating MASH by improving intestinal barrier function. C57BL/6J mice fed a choline-deficient amino acid-defined high-fat diet (CDA-HFD) were orally treated with B-mEVs, and therapeutic effects were evaluated. Liver histology, fibrosis, portal lipopolysaccharide (LPS) levels, intestinal permeability, and gut microbiota composition were evaluated. The direct effects of B-mEVs on intestinal barrier function were assessed using palmitic acid-stimulated Caco-2 cells. Small RNA sequencing and microRNA enrichment analyses were performed to characterize B-mEV cargo. B-mEV treatment attenuated hepatic steatosis, inflammation, and fibrosis in CDA-HFD-fed mice and reduced serum aminotransferase levels, portal LPS concentrations, hepatic macrophage accumulation, and hepatic TLR4/NF-κB signaling activation. Meanwhile, B-mEVs restored intestinal tight junction proteins, including ZO-1, occludin, and claudin-1, and improved intestinal permeability in vivo. In Caco-2 cells, B-mEVs attenuated palmitic acid-induced barrier dysfunction and suppressed myosin light chain kinase expression. Gut microbiota analysis revealed partial restoration of *Akkermansia* abundance after B-mEV administration. Furthermore, to explore the molecular basis of these protective effects, small RNA sequencing demonstrated enrichment of regulatory microRNAs, including let-7a-5p, and pathway analyses identified associations with intestinal barrier and inflammatory signaling pathways. B-mEVs ameliorated CDA-HFD-induced steatohepatitis by restoring intestinal barrier integrity and suppressing gut-derived LPS/TLR4 inflammatory signaling. These findings suggested that milk EVs may represent a novel gut–liver axis-targeted therapeutic strategy for MASH.

## 1. Introduction

Metabolic dysfunction-associated steatohepatitis (MASH) is a progressive chronic liver disease that can lead to advanced fibrosis, cirrhosis, and hepatocellular carcinoma and has substantially increased in global prevalence in recent years. Among the pathological features of MASH, hepatic fibrosis is the most important determinant of liver-related prognosis, highlighting the need to elucidate the underlying mechanisms and develop novel therapeutic strategies [1,2,3].

Hepatic fibrosis develops as a consequence of persistent hepatocellular injury and chronic inflammation during MASH progression. Continuous inflammatory signaling activates hepatic stellate cells, resulting in excessive extracellular matrix deposition and progressive fibrosis. Therefore, interventions that attenuate hepatic inflammation may also prevent the development and progression of liver fibrosis. Accordingly, therapeutic strategies targeting chronic hepatic inflammation may suppress not only steatohepatitis but also subsequent hepatic fibrogenesis [4].

Accumulating evidence has demonstrated that disruption of the gut–liver axis plays a critical role in MASH progression. Increased intestinal permeability facilitates translocation of gut-derived lipopolysaccharide (LPS) into the portal circulation, leading to activation of Kupffer cells and subsequent TLR4/NF-κB-mediated inflammatory signaling in the liver. Persistent activation of this inflammatory cascade further stimulates hepatic stellate cells, thereby linking intestinal barrier dysfunction with both steatohepatitis and hepatic fibrogenesis. Consequently, restoration of intestinal barrier integrity and gut microbial homeostasis has emerged as a promising therapeutic strategy for improving both inflammatory and fibrotic liver injury [5,6,7].

Extracellular vesicles (EVs) are nano-sized particles that mediate intercellular communication by transferring bioactive molecules, including microRNAs (miRNAs), proteins, and lipids. In particular, bovine milk-derived EVs (B-mEVs) have attracted increasing attention because of their relative stability within the gastrointestinal tract after oral administration and their potential biological activities in vivo [8,9,10]. In addition, miRNAs enriched in B-mEVs have been implicated in regulation of inflammatory responses, immune signaling, and intestinal homeostasis [11]. However, the effects of B-mEVs on MASH progression, especially regarding intestinal barrier dysfunction and gut–liver axis-associated hepatic fibrosis, remain poorly understood.

In this study, we investigated whether B-mEVs improve intestinal barrier integrity and suppress portal endotoxemia, thereby attenuating the progression of steatohepatitis and hepatic fibrosis through modulation of the gut–liver axis. We further evaluated alterations in gut microbiota composition and explored the molecular mechanisms underlying these protective effects using a CDA-HFD-induced murine MASH model, Caco-2 intestinal epithelial cells, and small RNA sequencing of B-mEVs.

## 2. Results

### 2.1. Bovine Milk-Derived Extracellular Vesicles Ameliorated Steatohepatitis and Hepatic Lipid Accumulation in CDA-HFD-Fed Mice

**Figure 1:**
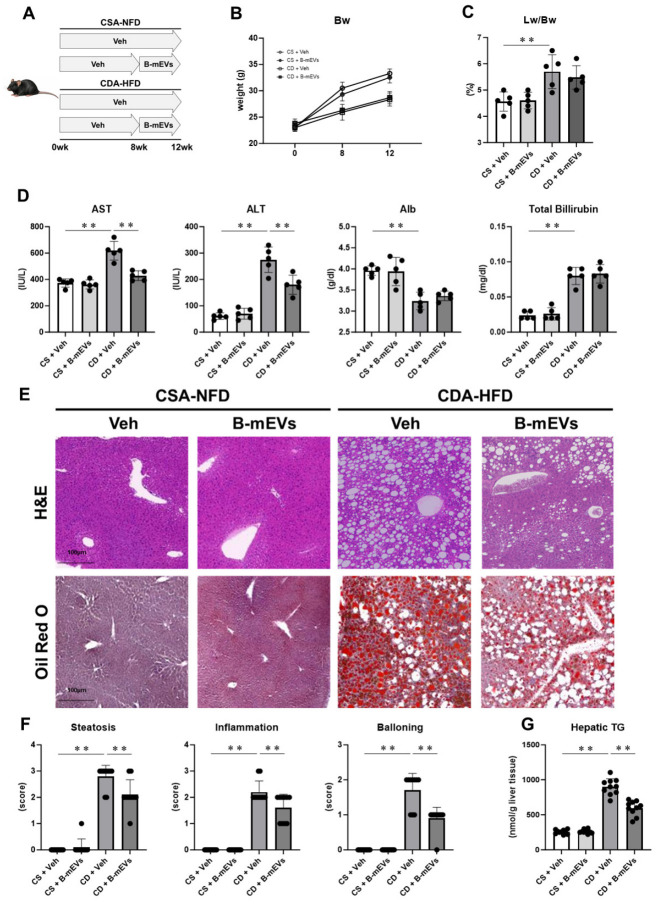
B-mEVs attenuate steatohepatitis and hepatic lipid accumulation in CDA-HFD-fed mice. (**A**) Schematic illustration of the experimental design. Mice were fed either a CSA-NFD or CDA-HFD diet for 12 weeks and treated with vehicle or B-mEVs on weeks 8–12. (**B**) Changes in body weight during the experimental period. (**C**) Relative liver weight at sacrifice (% of body weight). (**D**) Serum biochemical parameters, including AST, ALT, albumin, and total bilirubin levels. (**E**) Representative H&E and Oil Red O staining of liver sections. Scale bars, 100 µm. (**F**) Histological subscores for steatosis, inflammation, and hepatocellular ballooning. (**G**) Hepatic triglyceride content. Data are presented as mean ± SEM. ** *p* < 0.01. B-mEVs, bovine milk-derived extracellular vesicles; CDA-HFD, a choline-deficient amino acid-defined high-fat diet; CSA-NFD, a choline-supplemented L-amino acid-defined normal-fat diet; H&E, hematoxylin and eosin.

Compared with the a choline-supplemented L-amino acid-defined normal-fat diet (CSA-NFD) group, the CDA-HFD group had significantly reduced body weight and increased relative liver weight after 12 weeks; however, these changes were not significantly improved by B-mEV administration (Figure 1B,C). Serum AST and ALT levels significantly increased in the CDA-HFD-fed mice, and B-mEV treatment significantly attenuated this increase. In contrast, CDA-HFD-induced reductions in serum albumin levels and elevations in total bilirubin levels were not significantly improved by B-mEV administration (Figure 1D).

Histological analyses of H&E-stained liver sections demonstrated that CDA-HFD-fed mice developed typical MASH-like lesions characterized by marked macrovesicular steatosis, hepatocyte ballooning, and inflammatory cell infiltration, whereas these pathological changes were markedly attenuated following B-mEV treatment. Consistent with these histological findings, the NAFLD activity score was significantly reduced in the B-mEV-treated group. Oil Red O staining revealed extensive hepatic lipid droplet accumulation in the CDA-HFD group, which was markedly decreased following B-mEV administration. Furthermore, hepatic TG content was significantly lower in B-mEV-treated mice than in vehicle-treated CDA-HFD-fed mice (Figure 1E–G). Collectively, these findings demonstrate that B-mEV treatment ameliorated steatohepatitis and reduced hepatic lipid accumulation in CDA-HFD-fed mice.

### 2.2. B-mEVs Suppressed Hepatic Fibrosis Progression in CDA-HFD-Fed Mice

**Figure 2:**
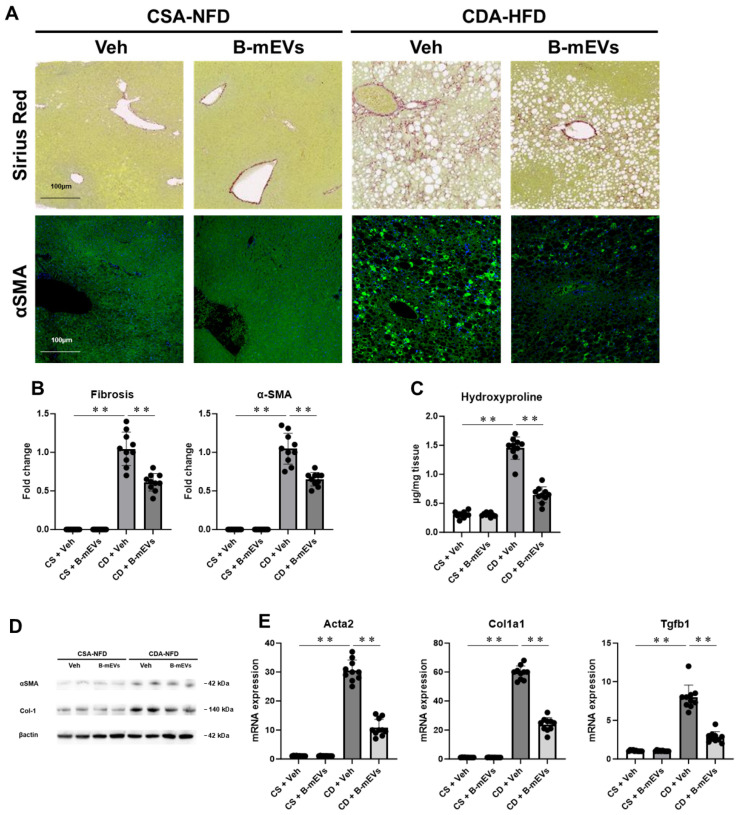
B-mEVs attenuate hepatic fibrosis in CDA-HFD-fed mice. (**A**) Representative Sirius Red staining and α-SMA immunostaining of liver sections. Scale bars, 100 µm. (**B**) Quantification of Sirius Red-positive and α-SMA-positive areas. (**C**) Hepatic hydroxyproline content. (**D**) Western blot analysis of fibrosis-related proteins, including COL1A1 and α-SMA, in liver tissues. (**E**) Hepatic mRNA expression levels of fibrogenic genes, including *Acta2*, *Col1a1*, and *Tgfb1*. Data are presented as mean ± SEM. ** *p* < 0.01. B-mEVs, bovine milk-derived extracellular vesicles; CDA-HFD, a choline-deficient amino acid-defined high-fat diet; α-SMA, α-smooth muscle actin; Col1a1, collagen type I alpha 1.

Representative Sirius Red staining demonstrated prominent pericellular fibrosis in CDA-HFD-fed mice, which was markedly attenuated by B-mEV treatment (Figure 2A). Consistently, enhanced α-SMA immunostaining indicated activation of hepatic stellate cells (HSCs) in the CDA-HFD group, whereas this was markedly reduced following B-mEV administration (Figure 2B).

Consistent with these histological findings, hepatic hydroxyproline content was significantly increased in CDA-HFD-fed mice and significantly decreased following B-mEV treatment (Figure 2C). In addition, the expression levels of fibrosis-related proteins, including collagen type I alpha 1 (COL1A1) and α-SMA, were significantly suppressed in the B-mEV-treated group (Figure 2D). Moreover, the hepatic mRNA expression of fibrosis-related genes, including Acta2, Col1a1, and Tgfb1, was significantly reduced by B-mEV administration (Figure 2E).

### 2.3. B-mEVs Reduced Portal LPS Levels and Suppressed Hepatic LPS/TLR4-Dependent Inflammatory Responses

**Figure 3:**
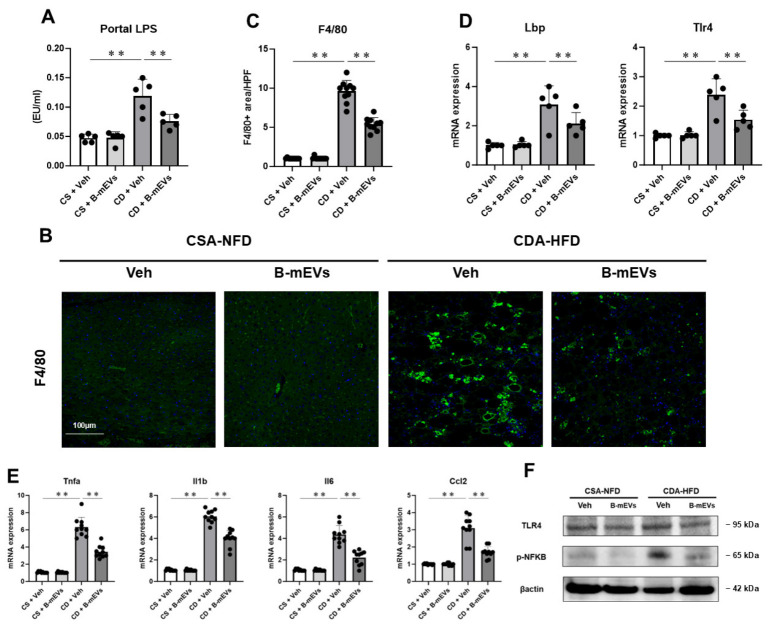
B-mEVs suppress portal endotoxemia and hepatic LPS/TLR4-mediated inflammatory signaling in CDA-HFD-fed mice. (**A**) Portal vein LPS levels. (**B**) Representative immunofluorescence staining for F4/80-positive hepatic macrophages. Scale bars, 100 µm. (**C**) Quantification of F4/80-positive areas. (**D**) Hepatic mRNA expression levels of *Lbp* and *Tlr4.* (**E**) Hepatic mRNA expression levels of inflammatory cytokines and chemokines, including *Tnf*, *Il1b*, *Il6*, and *Ccl2*. (**F**) Western blot analysis of TLR4 and p-NF-κB expression in liver tissues. B-mEVs, bovine milk-derived extracellular vesicles; LPS, lipopolysaccharide; TLR4, Toll-like receptor 4; p-NF-κB, phosphorylated nuclear factor kappa B. Data are presented as mean ± SEM. ** *p* < 0.01.

Gut-derived LPS is known to play a critical role in the progression of hepatic inflammation and fibrosis through the gut–liver axis [12]. Therefore, we investigated the effects of B-mEVs on portal LPS influx and LPS/TLR4-dependent hepatic inflammation. Compared with CSA-NFD-fed mice, CDA-HFD-fed mice had significantly higher portal venous LPS levels, and B-mEV administration significantly suppressed this increase (Figure 3A). Consistent with this finding, accumulation of F4/80-positive hepatic macrophages, including Kupffer cells, in CDA-HFD-fed mice was markedly reduced by B-mEV treatment (Figure 3B,C). Lipopolysaccharide-binding protein (LBP) facilitates inflammatory signaling by transferring LPS to the CD14/TLR4 complex. Accordingly, B-mEV treatment significantly decreased the hepatic mRNA expression of Lbp and Tlr4 (Figure 3D). Furthermore, consistent with suppression of LPS/TLR4 signaling, B-mEVs significantly reduced the hepatic expression of inflammatory cytokine and chemokine genes, including Tnfa, Il1b, Il6, and Ccl2 (Figure 3E). Western blot analysis demonstrated increased hepatic TLR4 expression and NF-κB phosphorylation in CDA-HFD-fed mice, both of which were attenuated by B-mEV administration (Figure 3F). These findings suggested that B-mEVs suppress hepatic TLR4/NF-κB-dependent inflammatory signaling by reducing portal LPS influx.

### 2.4. B-mEVs Ameliorated Intestinal Barrier Dysfunction in CDA-HFD-Fed Mice

**Figure 4:**
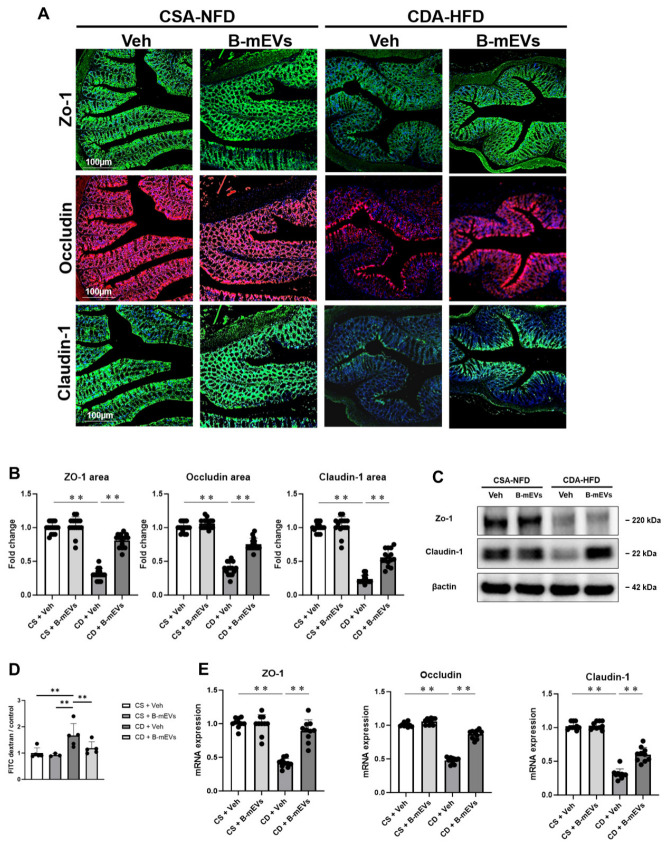
B-mEVs improve intestinal barrier dysfunction in CDA-HFD-fed mice. (**A**) Representative immunofluorescence staining for tight junction proteins, including ZO-1, occludin, and claudin-1, in intestinal tissues. Scale bars, 100 µm. (**B**) Quantification of fluorescence intensity of tight junction proteins. (**C**) Western blot analysis of ZO-1, occludin, and claudin-1 protein expressions in intestinal tissues. (**D**) Intestinal permeability assessed by FITC-dextran assay. (**E**) Intestinal mRNA expression levels of *Zo-1*, *Occludin*, and *Cldn1*. B-mEVs, bovine milk-derived extracellular vesicles; CDA-HFD, a choline-deficient amino acid-defined high-fat diet. Data are presented as mean ± SEM. ** *p* < 0.01.

We evaluated intestinal barrier function to investigate the mechanism of B-mEV-induced reduction in portal LPS levels. Figure 4 shows the effects of B-mEVs on intestinal barrier dysfunction in CDA-HFD-fed mice. Immunofluorescence analyses demonstrated marked reductions in the intestinal epithelial expression of the tight junction-associated proteins ZO-1, occludin, and claudin-1 in CDA-HFD-fed mice. B-mEV treatment restored both the expression and continuous localization of these tight junction proteins along the intestinal epithelium (Figure 4A). Consistent with these findings, Western blot analyses demonstrated that B-mEV administration restored the protein expression of ZO-1, occludin, and claudin-1 in intestinal tissues (Figure 4B,C). Moreover, CDA-HFD-fed mice exhibited significantly increased portal FITC-dextran levels, indicating enhanced intestinal permeability, and this increase was markedly attenuated by B-mEV treatment (Figure 4D). In addition, intestinal mRNA expression levels of Zo-1, Occludin, and Cldn1 were significantly increased in B-mEV-treated mice (Figure 4E).

### 2.5. B-mEVs Directly Ameliorated Palmitic Acid-Induced Barrier Dysfunction in Intestinal Epithelial Cells

**Figure 5:**
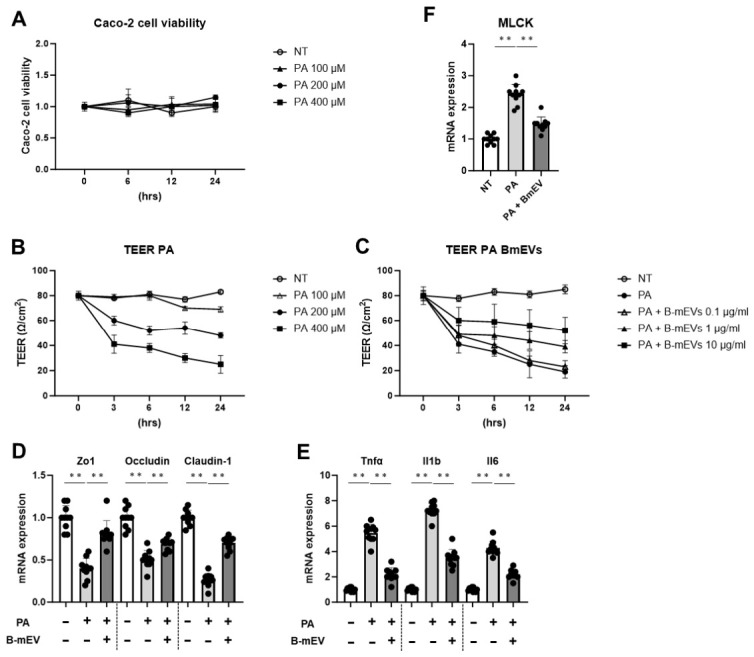
B-mEVs directly protect against PA-induced barrier dysfunction in Caco-2 intestinal epithelial cells. (**A**) Cell viability after PA treatment at different concentrations. (**B**) TEER values after PA stimulation. (**C**) Effects of B-mEVs on PA-induced reductions in TEER values. (**D**) Western blot analysis of ZO-1, occludin, and claudin-1 protein expressions after PA and B-mEV treatment. (**E**) mRNA expression levels of inflammatory cytokines. (**F**) MLCK expression after PA and B-mEV treatment. B-mEVs, bovine milk-derived extracellular vesicles; PA, palmitic acid; TEER, transepithelial electrical resistance. Data are presented as mean ± SEM. ** *p* < 0.01.

As shown in Figure 5A,B, PA stimulation at concentrations ranging from 100 to 400 μM dose-dependently reduced TEER values, an indicator of epithelial barrier integrity, without significantly affecting cell viability. B-mEV treatment dose-dependently attenuated the PA-induced reduction in TEER values (Figure 5C). These findings suggested that, under the present experimental conditions, PA-induced barrier dysfunction occurred independently of overt cell death and that B-mEVs exerted direct barrier-protective effects on intestinal epithelial cells. Consistent with these findings, B-mEVs restored the protein expression of ZO-1, occludin, and claudin-1, which had been reduced by PA stimulation (Figure 5D). Furthermore, B-mEV treatment significantly suppressed the PA-induced upregulation of inflammatory cytokine expression (Figure 5E). Interestingly, PA stimulation increased myosin light chain kinase (MLCK) expression in Caco-2 cells, and this increase was attenuated by B-mEV treatment (Figure 5F). These findings suggest that B-mEVs directly improve intestinal epithelial barrier dysfunction through MLCK-related signaling pathways.

### 2.6. B-mEVs Partially Restored Gut Microbiota Dysbiosis in CDA-HFD-Fed Mice

16S rRNA gene amplicon sequencing generated analyzable paired-end FASTQ data from all 12 samples. A total of 7,784,077 paired-end read pairs were obtained; after quality control and taxonomic annotation using the QIIME2 pipeline, 5,598,417 clean tags were retained for downstream analyses. These results confirmed sufficient sequencing depth for microbiome composition analysis and intergroup comparisons.

**Figure 6:**
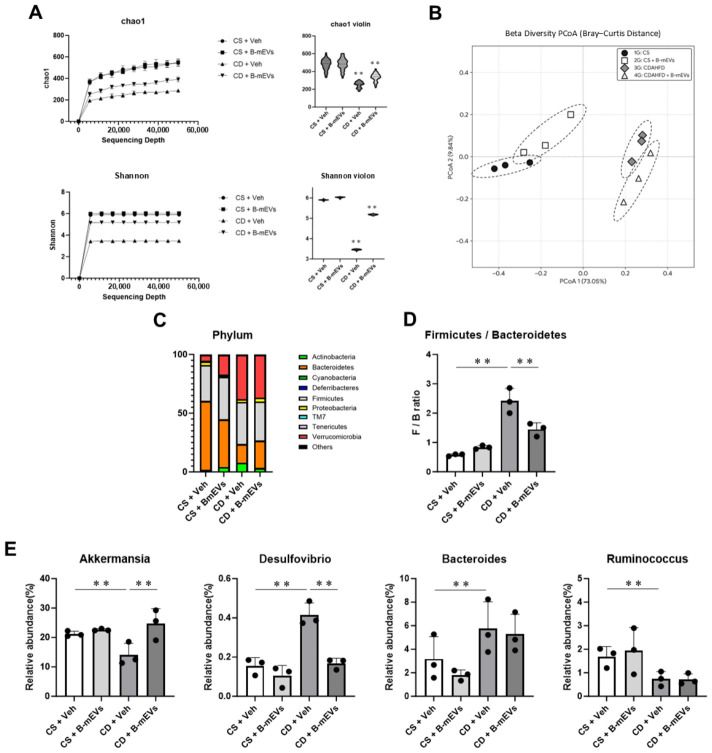
B-mEVs partially restore gut microbial dysbiosis in CDA-HFD-fed mice. (**A**) Alpha diversity analysis of fecal microbiota based on Chao1 and Shannon indices. (**B**) PCoA based on weighted UniFrac distances shows β diversity among groups. (**C**) Relative abundance of fecal microbiota at the phylum level. (**D**) *Firmicutes*/*Bacteroidetes* ratio in each group. (**E**) Relative abundance of representative bacterial genera associated with intestinal barrier function, including *Akkermansia*, *Desulfovibrio*, *Bacteroides*, and *Ruminococcus.* B-mEVs, bovine milk-derived extracellular vesicles; CDA-HFD, a choline-deficient amino acid-defined high-fat diet; PCoA, principal coordinate analysis. Data are presented as mean ± SEM. ** *p* < 0.01.

Alpha diversity analyses demonstrated significant reductions in both the Chao1 and Shannon indices in CDA-HFD-fed mice, compared with those in CSA-NFD-fed mice. B-mEV treatment partially restored the reduced alpha diversity induced by CDA-HFD feeding (Figure 6A). Beta diversity analysis based on weighted UniFrac distances demonstrated clear separation between the CDA-HFD + vehicle and CSA-NFD groups on principal coordinate analysis. In contrast, the overall effects of B-mEV administration on microbial community structure were relatively modest (Figure 6B). These findings suggested that B-mEVs may partially improve intestinal microbial homeostasis rather than broadly altering gut microbiota composition.

At the phylum level, the relative abundance of Firmicutes and Bacteroidetes was altered in CDA-HFD-fed mice (Figure 6C). Consequently, the Firmicutes/Bacteroidetes ratio was significantly increased compared with that in CSA-NFD-fed mice, whereas B-mEV treatment attenuated this alteration (Figure 6D). At the genus level, CDA-HFD feeding induced significant alterations in 11 taxa (unadjusted *p* < 0.05), including increases in 7 taxa and decreases in 4 taxa. Under CDA-HFD conditions, B-mEV treatment significantly altered two of these taxa; however, no taxon change remained significant after multiple testing correction.

Among these taxa, we focused on *Akkermansia*, *Desulfovibrio*, *Bacteroides*, and *Ruminococcus* because these genera have been implicated in intestinal barrier regulation [13]. Under CDA-HFD conditions, the relative abundance of *Akkermansia* was significantly decreased, whereas those of *Desulfovibrio* and *Bacteroides* were increased (Figure 6E). B-mEV treatment partially restored the abundance of *Akkermansia* and *Desulfovibrio*, whereas no apparent changes were observed for *Bacteroides* or *Ruminococcus* (Figure 6E).

### 2.7. miRNA Profiling and Functional Enrichment Analysis of B-mEVs

Small RNA sequencing analysis of B-mEVs analyzed 1,113,838,839 input reads and generated 58,323,296 unique molecular identifier (UMI) groups after filtering. These were integrated into 28,069,000 merged UMI groups, of which 10,057,793 were successfully annotated. Specifically, 5,768,843 UMI groups were assigned to miRBase *Bos taurus* miRNAs, whereas 4,288,950 UMI groups were assigned to Ensembl *Bos taurus* noncoding RNAs. In total, 371 mature miRNAs were identified in the milk exosome samples.

**Figure 7:**
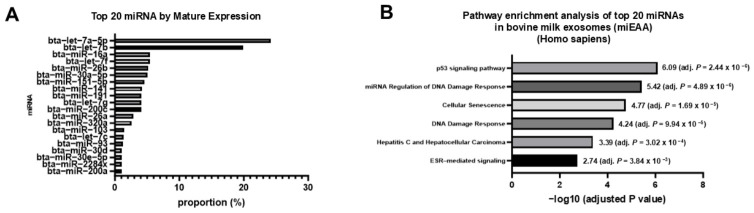
microRNA profiling and functional enrichment analysis of B-mEVs. (**A**) Small RNA sequencing identified these top 20 most abundant mature microRNAs in B-mEVs. Read counts were normalized to total exosomal small RNA reads. (**B**) Functional enrichment analysis of B-mEV-associated miRNAs using miRNA Enrichment Analysis and Annotation Tool 2.0 (miEAA 2.0) shows these representative significantly enriched pathways related to epithelial barrier regulation, inflammatory signaling, immune responses, and cellular homeostasis. Enrichment analysis was performed using human mature miRNA orthologs corresponding to bovine miRNAs. Statistical significance was determined using Benjamini–Hochberg false discovery rate correction. B-mEVs, bovine milk-derived extracellular vesicles.

The comprehensive expression profiles of the detected miRNAs are shown in Supplementary Figure S1A. Several miRNAs were highly abundant in B-mEVs, and the top 20 most abundant miRNAs are presented in Figure 7A. These miRNAs included milk EV-associated miRNAs that were previously reported to be involved in intestinal homeostasis, inflammatory regulation, and intercellular signaling [13]. Furthermore, functional enrichment analysis of the identified miRNAs was performed using the miRNA Enrichment Analysis and Annotation Tool 2.0 (miEAA 2.0). Supplementary Figure S1B presents the comprehensive functional enrichment analysis results, including the Gene Ontology Biological Process, Gene Ontology Cellular Component, KEGG, Reactome, and miRPathDB categories. The representative pathways potentially associated with intestinal barrier regulation and inflammatory responses are shown in Figure 7B. Gene Ontology Biological Process analysis demonstrated significant enrichment of pathways associated with epithelial barrier regulation, inflammatory responses, cell adhesion, and immune regulation. In addition, Gene Ontology Cellular Component analysis identified enrichment of categories related to cell junctions, EVs, and membrane-associated complexes. Moreover, KEGG, Reactome, and miRPathDB analyses revealed enrichment of signaling pathways associated with tight junctions, TLR signaling, NF-κB signaling, and intestinal homeostasis (Figure 7B). In addition, word cloud visualization highlighted the most significantly enriched functional categories identified by miEAA 2.0 (Supplementary Figure S1C).

These findings suggested that miRNAs contained in B-mEVs may contribute to regulation of intestinal barrier integrity and inflammatory responses relevant to MASH pathogenesis.

## 3. Discussion

In this study using a CDA-HFD-induced MASH model, we demonstrated that B-mEVs ameliorated steatohepatitis, hepatic lipid accumulation, and hepatic fibrosis; reduced portal LPS levels; suppressed TLR4/NF-κB-dependent inflammatory signaling; restored tight junction-associated protein expressions in the intestinal epithelium; and improved FITC-dextran permeability, suggesting amelioration of intestinal barrier dysfunction. In vitro analyses further demonstrated that B-mEVs directly attenuated PA-induced intestinal epithelial barrier dysfunction. Moreover, gut microbiota analysis revealed partial restoration of bacteria associated with intestinal homeostasis following B-mEV administration, and small RNA sequencing analysis demonstrated that miRNAs enriched in B-mEVs were associated with pathways related to intestinal barrier regulation and inflammatory responses. Collectively, these results indicate that B-mEVs primarily exert their protective effects through preservation of intestinal barrier integrity, thereby suppressing gut-derived inflammatory signaling and ultimately attenuating MASH progression.

Recently, increasing attention has been focused on the importance of the gut–liver axis in MASH pathogenesis. In particular, increased intestinal permeability and subsequent portal influx of LPS are known to activate TLR4/NF-κB signaling in Kupffer cells, thereby, promoting hepatic inflammation and fibrosis [12,14,15]. In this study, B-mEV administration reduced portal LPS levels, decreased hepatic *Tlr4* expression, and suppressed NF-κB activation. Our result indicates that B-mEVs may attenuate MASH progression by improving intestinal barrier function and subsequently suppressing LPS-dependent hepatic inflammatory signaling. These findings collectively suggest that preservation of intestinal barrier integrity is the primary event underlying the therapeutic effects of B-mEVs, whereas modulation of gut microbiota and miRNA-mediated signaling may act as complementary mechanisms.

In this study, B-mEV treatment restored the expressions of ZO-1, occludin, and claudin-1 in the mouse model and attenuated PA-induced TEER reductions and suppressed MLCK upregulation in Caco-2 cells. Activation of MLCK has been reported to increase intestinal permeability through disruption of tight junctions [16,17]. MLCK plays a pivotal role in the regulation of intestinal epithelial barrier integrity by phosphorylating myosin light chain, which promotes contraction of the perijunctional actomyosin ring and disrupts tight junction organization, thereby increasing intestinal permeability. In MASH and other inflammatory disorders, enhanced MLCK activity has been implicated in gut barrier dysfunction and subsequent translocation of gut-derived endotoxins into the portal circulation. In the present study, B-mEV treatment attenuated PA-induced MLCK expression together with restoration of tight junction proteins, suggesting that suppression of MLCK may contribute to the barrier-protective effects of B-mEVs. Overall, these observations support the possibility that B-mEVs directly protect intestinal epithelial barrier integrity through modulation of MLCK-related signaling pathways, which may represent an important upstream event in the gut–liver axis.

Gut microbiota analysis demonstrated that the overall effects of B-mEV treatment on microbial community structure were relatively modest, and reduction in *Akkermansia* abundance was partially restored. *Akkermansia muciniphila* is recognized as a representative bacterium involved in maintaining intestinal barrier integrity through regulation of the mucus layer and tight junctions [18,19].

Recently, the miRNA *let-7a-5p*, which is abundantly enriched in bovine colostrum-derived EVs, has been reported to ameliorate intestinal inflammation through modulation of *Akkermansia* abundance and intestinal metabolic homeostasis [20]. In that study, EV administration increased *Akkermansia* abundance and β-hydroxybutyrate production, and anti-inflammatory effects were replicated by administration of an *let-7a-5p* mimic. Consistent with these findings, this study showed highly abundant *let-7a-5p* in B-mEVs, which partially restored the reduction in *Akkermansia* abundance. These observations raised the possibility that B-mEV-derived miRNAs, particularly *let-7a-5p*, may influence host intestinal environment and/or gut microbiota composition, thereby contributing to maintenance of intestinal barrier homeostasis through *Akkermansia*-associated mechanisms. However, this study did not directly demonstrate a causal relationship between specific miRNAs and alterations in *Akkermansia* abundance. Further mechanistic investigations are warranted. Taken together, these findings suggest that modulation of specific gut microbes may contribute to, rather than solely account for, the barrier-protective effects of B-mEVs.

Small RNA sequencing analysis demonstrated that B-mEVs contained a large number of mature miRNAs, and functional enrichment analysis revealed associations with pathways related to tight junctions, TLR signaling, NF-κB signaling, and immune regulation. Previous studies have reported the involvement of miRNAs contained in milk-derived exosomes in inflammatory regulation, immune modulation, and intercellular communication [13,21]. Our findings further support the possibility that miRNAs enriched in B-mEVs may contribute to amelioration of MASH pathogenesis through regulation of intestinal barrier integrity and inflammatory responses. Together, these findings suggest that B-mEVs preserve intestinal barrier integrity through coordinated regulation of epithelial signaling and gut microbiota, while miRNA-mediated pathways may also contribute to these protective effects, thereby reducing portal endotoxemia, suppressing hepatic inflammation, and attenuating liver fibrosis in MASH. To our knowledge, this study provides proof-of-concept evidence that bovine milk-derived extracellular vesicles ameliorate experimental MASH through preservation of intestinal barrier integrity, suppression of portal endotoxemia, and modulation of the gut–liver axis. Further validation in larger preclinical studies will be required.

Several limitations of this study should be acknowledged. First, although the commercial B-mEV preparation was accompanied by manufacturer-provided quality control data, including nanoparticle tracking analysis, CD81 Western blotting, and a lot-specific certificate of analysis, additional characterization according to the MISEV2018 recommendations (e.g., CD63, TSG101, and non-EV markers) was not independently performed in the present study. Second, the number of animals was relatively limited, particularly in the gut microbiota analysis. Third, the causal relationship between microbiota alterations and intestinal barrier function improvement was not fully elucidated. In particular, the direct contribution of B-mEV-induced alterations in *Akkermansia* abundance to MASH amelioration remains unclear. Further studies using fecal microbiota transplantation and germ-free mouse models are required. Fourth, the functional roles of individual miRNAs contained in B-mEVs were not directly validated. Likewise, although B-mEV treatment was associated with suppression of MLCK expression, the causal role of MLCK in mediating the barrier-protective effects of B-mEVs was not directly investigated. Future studies employing gain- and loss-of-function approaches for candidate miRNAs, including let-7a-5p, as well as pharmacological or genetic modulation of MLCK, are warranted to clarify their mechanistic contributions. In addition, only female mice were used to maintain consistency with our experimental model. Future studies including both sexes are warranted. Finally, although the CDA-HFD model reproduced several pathological features of human MASH, it did not fully represent obesity or insulin resistance [22]. Therefore, validation using additional metabolic MASH models will be necessary.

## 4. Materials and Methods

### 4.1. Animal Experiments

All procedures were conducted over a 12-week period. Female C57BL/6J mice (*n* = 20, 7 weeks old; CLEA Japan, Osaka, Japan) were housed under controlled conditions (temperature: 23 ± 3 °C; humidity: 50 ± 20%; 12 h light/dark cycle). The mice were randomly assigned to the following four groups (*n* = 5 per group), according to the diets received for 12 weeks: (1) CSA-NFD (Research Diets Inc., New Brunswick, NJ, USA) with vehicle; (2) CSA-NFD with B-mEVs; (3) CDA-HFD (Research Diets Inc., New Brunswick, NJ, USA) with vehicle; and (4) CDA-HFD with B-mEVs. B-mEVs (1.2 mg/kg/day; Cosmo Bio Co., Ltd., Tokyo, Japan) [23] were administered by oral gavage every other day from weeks 8 to 12. The in vivo experimental design is shown in Figure 1A. Lactose hydrate (FUJIFILM Wako Pure Chemical Corporation, Osaka, Japan) was used as an excipient in the B-mEV preparation and was administered as the vehicle control in the relevant groups.

Bovine milk-derived extracellular vesicles (B-mEVs) were purchased from Cosmo Bio Co., Ltd. (Tokyo, Japan; Cat. Nos. EXBM100L/EXBM1000L). According to the manufacturer, B-mEVs were isolated from raw bovine milk by sequential ultracentrifugation (175,000× *g* for 70 min, three cycles) following defatting, whey preparation, and 0.22 μm filtration. The final preparation was filtered through a 0.22 μm membrane and supplied at a protein concentration of 100 μg/mL. Quality control performed by the manufacturer included nanoparticle tracking analysis (NanoSight LM10, Malvern Panalytical Ltd., Malvern, UK) and Western blotting for the EV marker CD81. The certificate of analysis for the lot used in this study reported a mean particle diameter of 198.9 nm and a particle concentration of 1.34 × 10^12^ particles/mL. Representative characterization data and the lot-specific certificate of analysis are provided in the Supplementary Materials.

After 12 weeks, the mice were anesthetized, and laparotomy was performed to collect blood samples from the aorta and portal vein. Liver and ileal tissues were excised and immediately fixed in neutral-buffered formalin for histological evaluation. This study was reviewed and approved by the ethics committee of Nara Medical University (No. 13501) and was performed in accordance with the Guide for Care and Use of Laboratory Animals of the National Research Council.

### 4.2. Biochemical Analysis and Hydroxyproline

Serum levels of aspartate aminotransferase (AST), alanine aminotransferase (ALT), albumin, and bilirubin were measured using Mouse Aspartate Aminotransferase ELISA, Mouse Alanine Aminotransferase ELISA, Mouse Albumin ELISA (Abcam, Waltham, MA, USA), and QuantiChrom™ Bilirubin Assay (BioAssay Systems, Hayward, CA, USA) kits, respectively, according to the manufacturers’ protocols. Hepatic triglyceride (TG) content was measured using Triglyceride-Glo™ Assay (Promega, Madison, WI, USA). Hepatic hydroxyproline content was measured using a Hydroxyproline Assay Kit (Cell Biolabs, San Diego, CA, USA), according to the manufacturer’s instructions.

### 4.3. Histological Analyses

Mouse liver samples were fixed in 4% paraformaldehyde and processed into paraffin sections for hematoxylin and eosin and Sirius red staining. Two pathologists independently determined the pathological score to assess liver tissue steatosis, inflammation, and ballooning, as previously described [24]. Other liver specimens were fixed in 4% paraformaldehyde for 24 h, then placed in a cryomold with Tissue-Tek O.C.T compound (Sakura Finetek Japan, Tokyo, Japan) to prepare frozen sections for Oil Red O staining.

### 4.4. Immunohistochemical Analyses

Paraffin liver and ileum sections (4 μm) were prepared for immunofluorescence; incubated with primary antibody overnight, followed by the secondary antibody; and mounted with 4′,6-diamidino-2-phenylindole. The primary antibodies included α-smooth muscle actin (SMA), F4/80, zonula occludens-1 (ZO-1), occludin, and claudin-1 (Supplementary Table S1). The secondary antibodies included Alexa Fluor-conjugated secondary antibodies (1:200; Thermo Fisher Scientific, Waltham, MA, USA). Semiquantitative analysis was performed using ImageJ software 1.54k (National Institutes of Health, Bethesda, MD, USA).

### 4.5. Real-Time Quantitative Polymerase Chain Reaction

Total RNA was isolated from cells and mouse liver tissue using QIAzole and Qiagen RNeasy Mini Kits (Qiagen, Hilden, Germany), followed by reverse transcription into cDNA using PrimeScript RT Reagent Kit (TaKaRa, Tokyo, Japan). Subsequently, quantitative polymerase chain reaction was performed using Power SYBR Green PCR Master Mix (Thermo Fisher Scientific, Waltham, MA, USA) and the specified primers detailed in Supplementary Table S2. Relative gene expression was calculated using the 2^−ΔΔCt^ method to normalize the relative mRNA expression levels of the target genes to those of the control group and GAPDH concentration.

### 4.6. Western Blotting

Total protein extraction commenced with sample treatment using a mammalian protein extraction reagent and then mixing with a protease (Halt™) and phosphatase inhibitor cocktail (Thermo Fisher Scientific, Waltham, MA, USA). Equal quantities of liver tissues and cell-extracted proteins were separated using 10% sodium dodecyl-sulfate polyacrylamide gel electrophoresis and then transferred to a polyvinylidene difluoride membrane (Invitrolon™; Thermo Fisher Scientific, Waltham, MA, USA). The protein-loaded membranes were blocked with defatted milk (5%) at room temperature for 2 h and incubated overnight with primary antibodies (Supplementary Table S1) at 4 °C. The next day, the membranes were washed and incubated with a horseradish peroxidase-labeled secondary antibody for 1 h at room temperature. Finally, target bands were detected using chemiluminescence. The loading control was β-actin. All experiments were performed at least three times.

### 4.7. Fluorescein Isothiocyanate-Dextran Intestinal Permeability Assay

A 4 kDa fluorescein isothiocyanate (FITC)-dextran solution (Sigma-Aldrich, St. Louis, MO, USA) was used to assess intestinal permeability [25]. Briefly, after 6 h of fasting, the mice in each group were administered FITC-dextran by oral gavage at a dose of 400 mg/kg body weight in a total volume of 200 µL. One hour after FITC-dextran administration, blood samples were collected from the portal vein. To evaluate intestinal permeability, serum fluorescence intensity was measured using a fluorospectrometer (NanoDrop 3300; Thermo Scientific, Waltham, MA, USA) at excitation and emission wavelengths of 490 nm and 520 nm, respectively.

### 4.8. Fecal Microbiome Analyses

Fecal samples collected from the colon were stored at −80 °C until analysis. A total of 12 samples (*n* = 3 per group) were subjected to 16S rRNA gene amplicon sequencing analysis [26]. The experimental groups were defined as follows: Group 1 (1G), CSA-NFD diet with vehicle treatment; Group 2 (2G), CSA-NFD diet with milk exosome treatment; Group 3 (3G), CDA-HFD diet with vehicle treatment; and Group 4 (4G), CDA-HFD diet with milk exosome treatment. Detailed methods for DNA extraction, library preparation, sequencing, and bioinformatic analyses are described in the Supplementary Materials.

### 4.9. In Vitro Cell Culture

The human intestinal epithelial cell line Caco-2 (RIKEN BioResource Research Center, Tsukuba, Ibaraki, Japan) was cultured in Dulbecco’s modified Eagle’s medium supplemented with 10% fetal bovine serum, 1% penicillin–streptomycin, 1% nonessential amino acids, and 25 mmol/L glucose at 37 °C in a humidified atmosphere containing 5% carbon dioxide, as previously described [27]. After 10–20 passages, cells were incubated in a culture medium with palmitic acid (PA) (Sigma-Aldrich, St. Louis, MO, USA) to evaluate cell viability and transepithelial electrical resistance (TEER). Cells treated with 200-µM PA were incubated with B-mEVs at different concentrations (0, 0.1, 1, and 10 µg/mL) for 3, 6, 12, and 24 h.

### 4.10. Cell Viability Assay

In vitro cell viability was determined using Premix WST-1 Cell Proliferation Assay system (Takara Bio, Kusatsu, Japan), according to the manufacturer’s protocol. Cell viability was calculated as the value relative to the start of exposure to each agent.

### 4.11. Measurement of Transepithelial Electrical Resistance

To assess in vitro Caco-2 monolayer barrier function, we measured TEER using an electrical resistance system (Millicell-ERS^®^; Millipore Corporation, Bedford, MA, USA), as reported previously [28]. TEER was expressed in Ω/cm^2^ using the surface area of the Transwell insert.

### 4.12. miRNA Profiling of B-mEVs Using Small RNA Sequencing

Total RNA was extracted from milk exosome samples using MagMAX mirVana Total RNA Isolation Kit (Thermo Fisher Scientific, Waltham, MA, USA), according to the manufacturer’s instructions. Small RNA sequencing library preparation and sequencing analyses were performed by Takara Bio Inc. (Kusatsu, Shiga, Japan); detailed methods are described in the Supplementary Materials.

### 4.13. microRNA Functional Enrichment Analysis

To investigate the biological functions and signaling pathways associated with the miRNAs contained in B-mEVs, functional enrichment analysis was performed using miRNA Enrichment Analysis and Annotation Tool 2.0 (miEAA 2.0; https://ccb-compute2.cs.uni-saarland.de/mieaa, accessed 13 May 2026) [29]. Bovine miRNAs were converted to the corresponding human mature miRNA identification based on conserved miRNA family names and miRBase nomenclature [30]. Bovine miRNAs without clear human mature miRNA counterparts were excluded from analysis. The converted human mature miRNA list was subsequently subjected to overrepresentation analysis using *Homo sapiens* as the target species.

The following annotation categories were analyzed: Gene Ontology Biological Process, Gene Ontology Cellular Component, Kyoto Encyclopedia of Genes and Genomes (KEGG) pathways, Reactome pathways, and miRPathDB. Additional disease-associated categories were evaluated when necessary. The default background set provided by miEAA 2.0 was used for enrichment analysis. Multiple testing correction was performed using the Benjamini–Hochberg false discovery rate method, and adjusted *p* values < 0.05 were considered statistically significant.

### 4.14. Statistical Analyses

Data are presented as mean ± SD. Statistical significance was analyzed using two-sided Student’s *t*-test or one-way analysis of variance, followed by Bonferroni’s multiple comparison test, as appropriate, using GraphPad Prism version 9.0 (GraphPad Software, La Jolla, CA, USA). Statistical significance was defined as *p* < 0.05.

## 5. Conclusions

In conclusion, this study demonstrated that B-mEVs ameliorated MASH pathology by improving intestinal barrier dysfunction and suppressing portal LPS influx and TLR4/NF-κB-dependent inflammatory signaling. In addition, miRNAs enriched in B-mEVs and partial restoration of gut microbiota homeostasis may contribute to these beneficial effects. These findings suggested that B-mEVs may represent a novel therapeutic strategy targeting the gut–liver axis in MASH.

## Data Availability

The datasets generated and/or analyzed during the current study are available from the corresponding author upon reasonable request.

## Supplementary Materials

The following supporting information can be downloaded at: https://www.mdpi.com/article/10.3390/ijms27146485/s1.

## Abbreviations

The following abbreviations are used in this manuscript:

EV	extracellular vesicles
HSC	hepatic stellate cells
KEGG	Kyoto Encyclopedia of Genes and Genomes
LBP	Lipopolysaccharide-binding protein
MLCK	myosin light chain kinase
PA	palmitic acid
RA	relative abundance
TEER	transepithelial electrical resistance
UMI	unique molecular identifier
