# Supplementary material for: Bovine Milk-Derived Extracellular Vesicles Ameliorate Steatohepatitis by Restoring Gut Barrier in CDA-HFD-Fed Mice
Source: Int J Mol Sci. 2026 Jul 21;27(14):6485. doi: 10.3390/ijms27146485 (PMC13409874; doi:10.3390/ijms27146485)
Supplement: Supplementary file 1 [file ijms-27-06485-s001.zip › Bovine_Milk_Exosome_EXBM100_1000_EN.pdf]

General Research Kit

コスモ・バイオ株式会社
COSMO BIO CO., LTD.

Bovine Milk Exosome

Bovine Milk-Derived Exosome

Cat. No. EXBM100L, EXBM1000L

Prepared: November 25, 2024

[I] Background

Exosomes are membrane vesicles roughly 50–150 nm in diameter that are secreted by most cell types. In vivo, they have been observed in body fluids such as saliva, blood, urine, amniotic fluid, and malignant ascites, and their secretion from cultured cells has also been confirmed. Exosomes encapsulate miRNA, mRNA, proteins, and micropeptides, and are suggested to play a role in transmitting information from their cell or tissue of origin to distant target cells or tissues, although their target specificity has not yet been fully clarified. [1] On the other hand, it has been shown that neutralizing exosome-mediated signal transmission with antibodies specific to exosome surface antigens such as CD9 and CD63 can suppress cancer metastasis. [2]

Exosomes are also present in the milk of a wide range of animals, including humans, cattle, and rats. They encapsulate miRNA, mRNA, and proteins, and it has been suggested that these molecules function after being taken up by target cells. [3]

In addition, recent research has made use of these exosome properties to explore their potential as drug delivery system (DDS) carriers. In particular, bovine milk-derived exosomes have attracted attention as a means of oral administration for anti-cancer drugs. [4]

This product is an exosome fraction prepared by ultracentrifugation from raw milk collected from healthy cows, and can be used in a wide range of experiments from in vitro to in vivo. This product was developed by applying the results of a research project led by Dr. Yusuke Yoshioka, Division of Molecular and Cellular Medicine, National Cancer Center Research Institute.

Japan Agency for Medical Research and Development (AMED)
Basic Research Program for Innovative Biological Drug Discovery
Project title: Development of a novel drug delivery system using exosome engineering technology
Principal investigator: Yusuke Yoshioka

Cat. No.	Product Name	Packaging	Concentration	Applications	Storage
EXBM100L	Bovine Milk-Derived Exosome	100 µL × 10 tubes	Protein concentration: 100 µg/mL, PBS, sterile filtered	Administration studies, DDS studies, nucleic acid/protein extraction, etc.	4℃
EXBM1000L	Bovine Milk-Derived Exosome	1 mL × 10 tubes	Protein concentration: 100 µg/mL, PBS, sterile filtered	Administration studies, DDS studies, nucleic acid/protein extraction, etc.	4℃

** The exosome particle count varies by lot. Please refer to the Certificate of Analysis (CoA) included with the product.*

Cosmo Bio Co., Ltd. [Manufacturer code: CSR]

This product is for research use only. Do not use for medical treatment or clinical diagnosis in humans or animals.
Please read this manual carefully and use for research purposes only.

[II] Instructions for Use

Use the product immediately upon arrival, or store at 4℃.

Related Products

Cosmo Bio Co., Ltd. [Manufacturer code: CAC]

Cat. No.	Product Name	Packaging	Storage
SHI-EXO-M03	Anti CD81, Human (Mouse) — cross-reactive with Human, Bovine	100 µL (1 mg/mL)	-80℃

[III] Reference Data (EXBM100L, EXBM1000L)

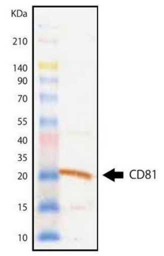

図 1 . CD81 モノクローナル抗体を用いた Western Blotting

タンパク質量として 2ug を用いて、Anti-CD81 モノクローナル抗体 (品番：SHI-EXO-M03) で検出した。

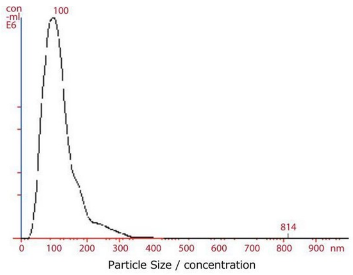

図 2. NanoSight を用いた粒度分布測定

Size Distribution: Mean: 119 nm

Figure 1. Western blotting using an anti-CD81 monoclonal antibody
Using 2 µg of protein, detection was performed with an anti-CD81 monoclonal antibody (Cat. No. SHI-EXO-M03).

Figure 2. Particle size distribution measured by NanoSight
Size distribution: Mean – 119 nm

[VI] References

[1] Tumour exosome integrins determine organotropic metastasis, Hoshino A, et al., Nature. 2015 Nov 19; 527(7578): 329-35.

[2] Disruption of Circulating Extracellular Vesicles as a Novel Therapeutic Strategy against Cancer Metastasis, Nao Nishida-Aoki, et al., Mol Ther. 2017 Jan 4; 25(1): 181-191.

[3] Bovine milk exosomes contain microRNA and mRNA and are taken up by human macrophages, Hirohisa Izumi, et al., J Dairy Sci. 2015 May; 98(5): 2920-33

[4] Milk-derived exosomes for oral delivery of paclitaxel, Ashish K. Agrawal et al., Nanomedicine. 2017 Jul; 13(5): 1627-1636.

コスモ・バイオ株式会社
COSMO BIO CO., LTD.

For pricing, stock, and delivery inquiries
TEL: 03-5632-9630 (Business hours: 9:00–17:30)
FAX: 03-5632-9623

For product inquiries
TEL: 03-5632-9610 (Business hours: 9:00–17:30)
FAX: 03-5632-9619

Head Office: Toyo Ekimae Bldg., 2-2-20 Toyo, Koto-ku, Tokyo 135-0016, Japan

This product is for research use only. Do not use for medical treatment or clinical diagnosis in humans or animals.
Please read this manual carefully and use for research purposes only.
