# Supplementary material for: Bovine Milk-Derived Extracellular Vesicles Ameliorate Steatohepatitis by Restoring Gut Barrier in CDA-HFD-Fed Mice
Source: Int J Mol Sci. 2026 Jul 21;27(14):6485. doi: 10.3390/ijms27146485 (PMC13409874; doi:10.3390/ijms27146485)
Supplement: Supplementary file 1 [file ijms-27-06485-s001.zip › Supplementary_materials.pdf]

Supplementary Table S1. List of primary antibodies

Antibody	Source (catalog number)	Application (Dilution)
α-SMA	Abcam (ab5694)	IHC (1:100)
F4/80	Abcam (ab111101)	IHC (1:100)
ZO-1	Invitrogen (61-7300)	IHC (1:200)
Occludin	Abcam (ab216327)	IHC (1:200)
Claudin1	Abcam (ab15098)	IHC (1:200)
β-Actin	Cell signaling (4967)	WB (1:1000)
α-SMA	Abcam (ab5694)	WB (1:1000)
Collagen I (Col-1)	Abcam (ab34710)	WB (1:1000)
TLR4	Abcam (ab13556)	WB (1:1000)
p-NF-κB p65	Cell Signaling Technology (#3033)	WB (1:1000)
ZO-1	Invitrogen (61-7300)	WB (1:1000)
Claudin-1	Abcam (ab15098)	WB (1:1000)

Supplementary Table S2. List of primers used in q-PCR

Gene	Sense (5′-3′)	Antisense (5′-3′)
Mouse		
Acta2	CTGACAGAGGCACCACTGAA	CATCTCCAGAGTCCAGCACA
Col1a1	GAGCGGAGAGTACTGGATCG	GCTTCTTTTCCTTGGGGTTC
Tgfb1	TTGCTTCAGCTCCACAGAGA	TGGTTGTAGAGGGCAAGGAC
Lbp	GGCTGCTGAATCTCTTCCAC	GAGCGGTGATTCCGATTAAA
Tlr4	GGCAGCAGGTGGAATTGTAT	AGGCCCCAGAGTTTTGTTCT
Tnfa	ACGGCATGGATCTCAAAGAC	AGATAGCAAATCGGCTGACG
Il1b	GCCCATCCTCTGTGACTCAT	AGGCCACAGGTATTTTGTCG
IL6	GAGCCCACCAAGAACGATAG	TCCACGATTTCCCAGAGAAC
Ccl2	AGGTCCCTGTCATGCTTCTG	TCTGGACCCATTCCTTCTTG
Zo1	GCTAAGAGCACAGCAATGGA	GCATGTTCAACGTTATCCAT
Ocln	ACTGGGTCAGGGAATATCCA	TCAGCAGCAGCCATGTACTC
Cldn1	TGCCCCAGTGGAAGATTTACT	CTTTGCGAAACGCAGGACAT
Gapdh	AGGTCGGTGTGAACGGATTTG	TGTAGACCATGTAGTTGAGGTCA
Human		
ZO1	CAACATACAGTGACGTTCACA	CACTATTGACGTTTCCCCACTC
OCLN	TCCTATAAATCCACGCCGGTTC	CTCAAAGTTACCACCGCTGCTG
CLDN1	CCTCCTGGGTTTCATCCTGG	CGAGCCGTACCTGGCATTG
TNFA	CCCAGGGACCTCTCTCTAATC	ATGGGCTACAGGCTTGTCACT
IL1B	ATGATGGCTTATTACAGTGGCAA	GTCGGAGATTCGTAGCTGGA
IL6	ACTCACCTCTTCAGAACGAATTG	CCATCTTTGGAAGGTTCAGGTTG
MYLK	GAGGAGATCGAGGCTGAGAA	CTGTTGGTGATGTTGGTGGT
GAPDH	GAAGGTGAAGGTCGGAGTC	GAAGATGGTGATGGGATTTC

Supplementary Figure S1. Comprehensive microRNA expression profiling and functional enrichment analysis of bovine milk-derived extracellular vesicles (B-mEVs).

(A) Comprehensive expression profile of mature microRNAs identified in B-mEVs by small RNA sequencing. Mature miRNAs are ranked according to normalized read counts.

(B) Comprehensive functional enrichment analysis of B-mEV-associated miRNAs performed using the miRNA Enrichment Analysis and Annotation Tool 2.0 (miEAA 2.0). Significantly enriched categories from Gene Ontology Biological Process, Gene Ontology Cellular Component, Kyoto Encyclopedia of Genes and Genomes (KEGG), Reactome, and miRPathDB are ranked according to significance (−log10 adjusted *p* value).

(C) Word cloud visualization of the significantly enriched functional categories identified by miEAA 2.0. The size of each term reflects its relative enrichment significance, with larger words representing more significantly enriched biological pathways or processes.

Fecal microbiome analyses

Genomic DNA was extracted from each fecal sample and used as a template for amplification of the V3–V4 region of the bacterial 16S rRNA gene using the 341F and 806R primer set. After purification of the PCR products, sequencing libraries were generated by adding unique index sequences to each sample. After library quantification, paired-end sequencing was performed using the Illumina NextSeq2000 platform. The obtained paired-end reads were processed using the QIIME2 pipeline (1). DADA2 (2) was used for denoising, removal of PCR primer sequences, paired-end read assembly, chimera detection and removal, and merging of identical sequences. Subsequently, clustering was performed using VSEARCH with 99% sequence similarity threshold to generate operational taxonomic unit (OTU) representative sequences. Taxonomic annotation of OTU representative sequences was performed using a scikit-learn Naive Bayes classifier trained on the GreenGenes 99% database and BLAST homology searches against the DDBJ 16S rRNA database. Relative abundance of each bacterial taxon was calculated based on clean tags at the phylum, class, order, family, genus, and species levels and then used for comparative analyses among groups. At the genus level (L6), the effect of CDA-HFD feeding was evaluated by comparing the CSA-NFD + vehicle group (1G) with the CDA-HFD + vehicle group (3G) (Set C). The effect of milk exosome treatment under CDA-HFD conditions was evaluated by comparing the CDA-HFD + vehicle group (3G) with the CDA-HFD + milk exosome group (4G) (Set B). Relative abundance of each bacterial taxon was compared using a nonparametric two-sample t-test.

miRNA profiling of B-mEVs by small RNA sequencing

Small RNA sequencing libraries were prepared using the QIAseq miRNA Library Kit and QIAseq

miRNA NGS 96 Index IL UDI v2, according to the QIAseq miRNA UDI Library Kit Handbook (August 2023) (3). Briefly, adapters were ligated to both ends of the RNA fragments, followed by reverse transcription using primers that recognized the RNA 3′ adapter. The resulting single-stranded cDNA was amplified by PCR using indexed primers, and PCR products were subsequently purified. After quality assessment of the purified PCR products, small RNA-sized libraries were selected using the BluePippin system. The prepared libraries showed a 192-bp peak size and a 12.0-nM concentration, which met the quality criteria required for sequencing analysis. Sequencing was performed on the Illumina NovaSeq X Plus platform using the NovaSeq X Series 10B Reagent Kit (4). Raw sequencing data were processed using Real Time Analysis (RTA) v4.6.7 and NovaSeqXSeries Control Software v1.2.0 and converted into FASTQ files using BCL Convert v4.3.6 (5). Sequencing generated 2,227,677,678 reads and 334,151,651,700 bases in total. The Q30 scores were 71.8% for read 1 and 58.7% for read 2.

For small RNA analysis, FASTQ data from read 1 were analyzed using CLC Genomics Workbench v20.0.4, Biomedical Genomics Analysis Plugin v20.2, and Python v3.6.8 (6). Reads were consolidated using a unique molecular identifier (UMI)-based workflow, and counts of mature miRNAs and other noncoding RNAs were calculated. Annotation was performed using miRBase release 22.1 (7) for miRNA and the Bos taurus ARS-UCD2.0 dataset from Ensembl release 114 (8) for other noncoding RNAs. Transcript abundance was quantified as raw counts and transcripts per million (TPM).

Supplementary References:

1. Bolyen, E.; Rideout, J.R.; Dillon, M.R.; Bokulich, N.A.; Abnet, C.C.; Al-Ghalith, G.A.; Alexander, H., Alm, E.J., Arumugam, M., Asnicar, F.; Bai, Y., Bisanz, J.E.; Bittinger, K.; Brejnrod, A.; Brislawn, C.J.; Brown, C.T.; Callahan, B.J.; Caraballo-Rodríguez, A.M.; Chase, J.; Cope, E.K.; Da Silva, R.; Diener, C.; Dorrestein, P.C.; Douglas, G.M.; Durall, D.M.; Duvallet, C.; Edwardson, C.F.; Ernst, M.; Estaki, M.; Fouquier, J.; Gauglitz, J.M.; Gibbons, S.M.; Gibson, D.L.; Gonzalez, A.; Gorlick, K.; Guo, J.; Hillmann, B.; Holmes, S.; Holste, H.; Huttenhower, C.; Huttley, G.A.; Janssen, S.; Jarmusch, A.K.; Jiang, L.; Kaehler, B.D.; Kang, K.B.; Keefe, C.R.; Keim, P.; Kelley, S.T.; Knights, D.; Koester, I.; Kosciolek, T.; Kreps, J.; Langille, M.G.I.; Lee, J.; Ley, R.; Liu, Y.-X.; Loftfield, E.; Lozupone, C.; Maher, M.; Marotz, C.; Martin, B.D.; McDonald, D.; McIver, L.J.; Melnik, A.V.; Metcalf, J.L.; Morgan, S.C.; Morton, J.T.; Naimey, A.T.; Navas-Molina, J.A.; Nothias, L.F.; Orchanian, S.B.; Pearson, T.; Peoples, S.L.; Petras, D.; Preuss, M.L.; Pruesse, E.; Rasmussen, L.B.; Rivers, A.; Robeson, M.S., II; Rosenthal, P.; Segata, N.; Shaffer, M.; Shiffer, A.; Sinha, R.;

Song, S.J.; Spear, J.R.; Swafford, A.D.; Thompson, L.R.; Torres, P.J.; Trinh, P.; Tripathi, A.; Turnbaugh, P.J.; Ul-Hasan, S.; van der Hooft, J.J.J.; Vargas, F.; Vázquez-Baeza, Y.; Vogtmann, E.; von Hippel, M.; Walters, W.; Wan, Y.; Wang, M.; Warren, J.; Weber, K.C.; Williamson, C.H.D.; Willis, A.D.; Xu, Z.Z.; Zaneveld, J.R.; Zhang, Y.; Zhu, Q.; Knight, R.; Caporaso, J.G.; Reproducible, interactive, scalable and extensible microbiome data science using QIIME 2. *Nat. Biotechnol.* **2019**, *37*, 852–857.

2. Callahan, B.J.; McMurdie, P.J.; Rosen, M.J.; Han, A.W.; Johnson, A.J.; Holmes, S.P. DADA2: High-resolution sample inference from Illumina amplicon data. *Nat. Methods* **2016**, *13*, 581–583.
3. QIAGEN. *QIAseq miRNA Library Kit Handbook*; QIAGEN: Hilden, Germany, 2023.
4. Bentley, D.R.; Balasubramanian, S.; Swerdlow, H.P.; Smith, G.P.; Milton, J.; Brown, C.G.; Hall, K.P.; Evers, D.J.; Barnes, C.L.; Bignell, H.R.; Boutell, J.M.; Bryant, J.; Carter, R.J.; Cheetham, R.K.; Cox, A.J.; Ellis, D.J.; Flatbush, M.R.; Gormley, N.A.; Humphray, S.J.; Irving, L.J.; Karbelashvili, M.S.; Kirk, S.M.; Li, H.; Liu, X.; Maisinger, K.S.; Murray, L.J.; Obradovic, B.; Ost, T.; Parkinson, M.L.; Pratt, M.R.; Rasolonjatovo, I.M.J.; Reed, M.T.; Rigatti, R.; Rodighiero, C.; Ross, M.T.; Sabot, A.; Sankar, S.V.; Scally, A.; Schroth, G.P.; Smith, M.E.; Smith, V.P.; Spiridou, A.; Torrance, P.E.; Tzonev, S.S.; Vermaas, E.H.; Walter, K.; Wu, X.; Zhang, L.; Alam, M.D.; Anastasi, C.; Aniebo, I.C.; Bailey, D.M.D.; Bancarz, I.R.; Banerjee, S.; Barbour, S.G.; Baybayan, P.A.; Benoit, V.A.; Benson, K.F.; Bevis, C.; Black, P.J.; Boodhun, A.; Brennan, J.S.; Bridgham, J.A.; Brown, R.C.; Brown, A.A.; Buermann, D.H.; Bundu, A.A.; Burrows, J.C.; Carter, N.P.; Castillo, N.; Catenazzi, M.C.E.; Chang, S.; Cooley, R.N.; Crake, N.R.; Dada, O.O.; Diakoumakos, K.D.; Dominguez-Fernandez, B.; Earnshaw, D.J.; Egbujor, U.C.; Elmore, D.W.; Etchin, S.S.; Ewan, M.R.; Fedurco, M.; Fraser, L.J.; Fuentes Fajardo, K.V.; Furey, W.S.; George, D.; Gietzen, K.J.; Goddard, C.P.; Golda, G.S.; Granieri, P.A.; Green, D.E.; Gustafson, D.L.; Hansen, N.F.; Harnish, K.; Haudenschild, C.D.; Heyer, N.I.; Hims, M.M.; Ho, J.T.; Horgan, A.M.; Hoschler, K.; Hurwitz, S.; Ivanov, D.V.; Johnson, M.Q.; James, T.; Jones, T.A.H.; Kang, G.-D.; Kerelska, T.H.; Kersey, A.D.; Khrebtukova, I.; Kindwall, A.P.; Kingsbury, Z.; Kokko-Gonzales, P.I.; Kumar, A.; Laurent, M.A.; Lawley, C.T.; Lee, S.E.; Lee, X.; Liao, A.K.; Loch, J.A.; Lok, M.; Luo, S.; Mammen, R.M.; Martin, J.W.; McCauley, P.G.; McNitt, P.; Mehta, P.; Moon, K.W.; Mullens, J.W.; Newington, T.; Ning, Z.; Ng, B.L.; Novo, S.M.; O'Neill, M.J.; Osborne, M.A.; Osnowski, A.; Ostadan, O.; Paraschos, L.L.; Pickering, L.; Pike, A.C.; Pike, A.C.; Pinkard, D.C.; Pliskin, D.P.; Podhasky, J.; Quijano, V.J.; Raczy, C.; Rae, V.H.; Rawlings, S.R.; Rodriguez, A.C.; Roe, P.M.; Rogers, J.; Rogert Bacigalupo, M.C.; Romanov, N.; Romieu, A.; Roth, R.K.; Rourke, N.J.; Ruediger, S.T.; Rusman, E.; Sanches-Kuiper, R.M.; Schenker, M.R.; Seoane, J.M.; Shaw, R.J.; Shiver, M.K.; Short, S.W.; Sizto, N.L.; Sluis,

J.P.; Smith, M.A.; Sohna Sohna, J.E.; Spence, E.J.; Stevens, K.; Sutton, N.; Szajkowski, L.; Tregidgo, C.L.; Turcatti, G.; vandeVondele, S.; Verhovsky, Y.; Virk, S.M.; Wakelin, S.; Walcott, G.C.; Wang, J.; Worsley, G.J.; Yan, J.; Yau, L.; Zuerlein, M.; Rogers, J.; Mullikin, J.C.; Hurles, M.E.; McCooke, N.J.; West, J.S.; Oaks, F.L.; Lundberg, P.L.; Klenerman, D.; Durbin, R.; Smith, A.J. Accurate whole human genome sequencing using reversible terminator chemistry. *Nature* **2008**, *456*, 53–59.
5. Illumina Inc. *BCL Convert Software Documentation*; Illumina: San Diego, CA, USA, 2023.
6. QIAGEN Digital Insights. *CLC Genomics Workbench 20.0 User Manual*; QIAGEN Digital Insights: Aarhus, Denmark, 2020.
7. Kozomara, A.; Birgaoanu, M.; Griffiths-Jones, S. miRBase: from microRNA sequences to function. *Nucleic Acids Res.* **2019**, *47*, D155–D162.
8. Cunningham, F.; Allen, J.E.; Allen, J.; Alvarez-Jarreta, J.; Amode, M.R.; Armean, I.M.; Austine-Orimoloye, O.; Azov, A.; Barnes, I.F.; Bennett, R.; Berry, A.; Bhai, J.; Bignell, A.; Billis, K.; Boddu, S.; Brooks, L.; Charkhchi, M.; Cummins, C.; Da Rin Fioretto, L.; Davidson, C.; Dodiya, K.; Donaldson, S.; El Houdaigui, B.; El Naboulsi, T.; Fatima, R.; Garcia Giron, C.; Genez, T.; Gonzalez Martinez, J.; Guijarro-Clarke, C.; Gymer, A.; Hardy, M.; Hollis, Z.; Hourlier, T.; Hunt, T.; Juettemann, T.; Kaikala, V.; Kay, M.; Lavidas, I.; Le, T.; Lemos, D.; Marugán, J.C.; Mohanan, S.; Mushtaq, A.; Naven, M.; Ogeh, D.N.; Parker, A.; Parton, A.; Perry, M.; Piližota, I.; Prosovetskaia, I.; Sakthivel, M.P.; Abdul Salam, A.I.; Schmitt, B.M.; Schuilenburg, H.; Sheppard, D.; Pérez-Silva, J.G.; Stark, W.; Steed, E.; Sutinen, K.; Sukumaran, R.; Sumathipala, D.; Suner, M.-M.; Szpak, M.; Thormann, A.; Tricomi, F.F.; Urbina-Gómez, D.; Veidenberg, A.; Walsh, T.A.; Walts, B.; Willhoft, N.; Winterbottom, A.; Wass, E.; Chakiachvili, M.; Flint, B.; Frankish, A.; Giorgetti, S.; Haggerty, L.; Hunt, S.E.; Ilsley, G.R.; Loveland, J.E.; Martin, F.J.; Moore, B.; Mudge, J.M.; Muffato, M.; Perry, E.; Ruffier, M.; Tate, J.; Thybert, D.; Trevanion, S.J.; Dyer, S.; Harrison, P.W.; Howe, K.L.; Yates, A.D.; Zerbino, D.R.; Flicek, P. Ensembl 2022. *Nucleic Acids Res.* **2022**, *50*, D988–D995.
