# Supplementary figures and images for: Bovine Milk-Derived Extracellular Vesicles Ameliorate Steatohepatitis by Restoring Gut Barrier in CDA-HFD-Fed Mice
Source: Int J Mol Sci. 2026 Jul 21;27(14):6485. doi: 10.3390/ijms27146485 (PMC13409874; doi:10.3390/ijms27146485)

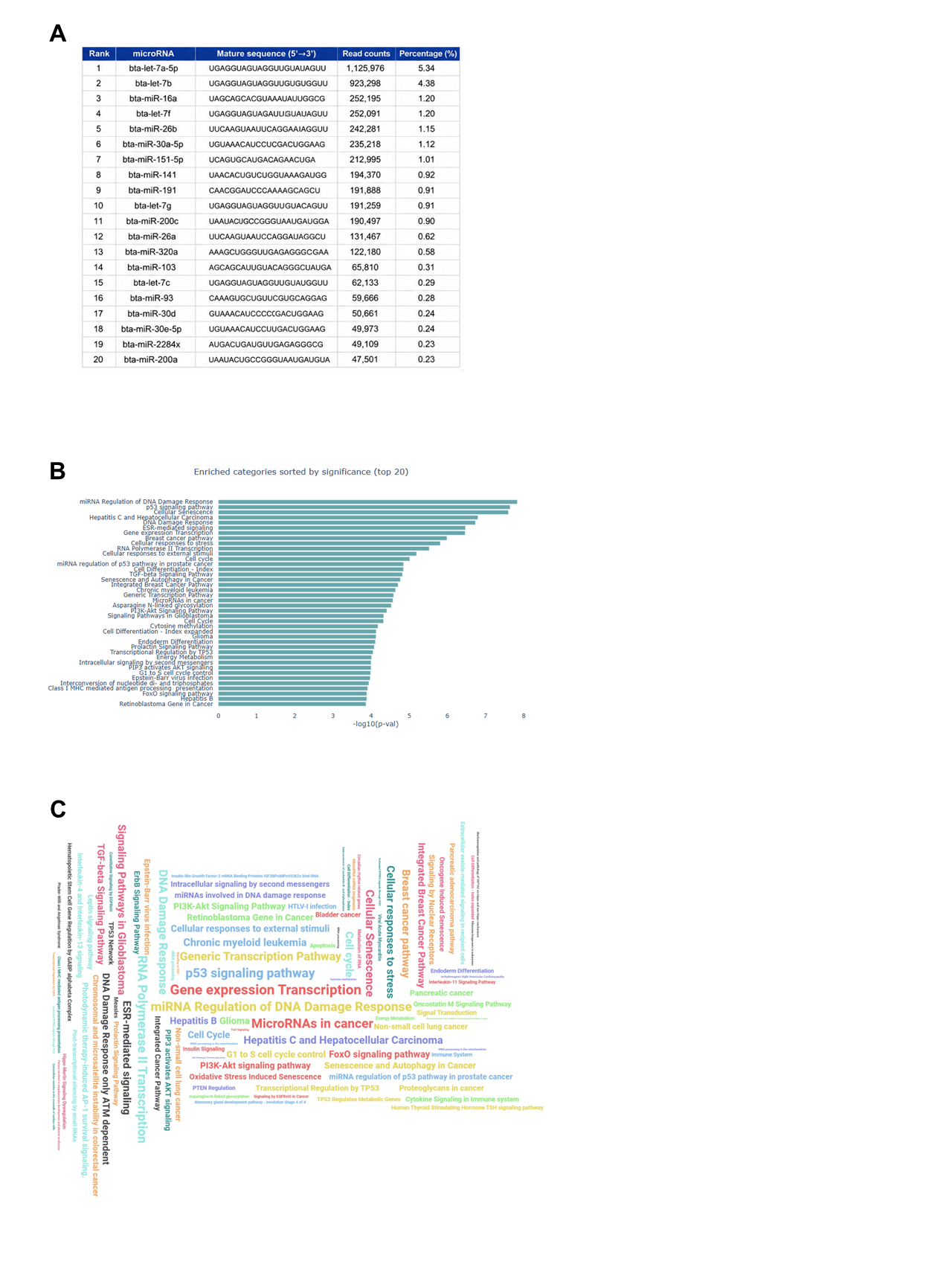

Supplement: Supplementary file 1 [file ijms-27-06485-s001.zip › Figure S1 PNG.png]
